# Exploring the impact of temperature and oxygen partial pressure on the spent nuclear fuel oxidation during its dry management

**DOI:** 10.1038/s41598-023-29265-w

**Published:** 2023-02-03

**Authors:** A. Milena-Pérez, L. J. Bonales, N. Rodríguez-Villagra, H. Galán

**Affiliations:** High-Level Waste Unit, Centro de Investigaciones Energéticas, Medioambientales y Tecnológicas (CIEMAT), Avda. Complutense 40, 28040 Madrid, Spain

**Keywords:** Corrosion, Energy

## Abstract

The management of Spent Nuclear Fuel (SNF) comprises different stages in which security is demonstrated. Nevertheless, fundamental research can lead to other design options that must be considered. Currently, one of the focuses is the dry interim storage option, as the shortest-term solution until final repositories are available. During this stage, one concern is the oxidation of the fuel. If UO_2_ (SNF matrix) is exposed to air at high-enough temperature, formation of U_3_O_8_ takes place. The larger volume of this phase could entail stresses on the SNF clad, which is the first barrier to prevent radioactive material release. It is known that this oxidation is a temperature-dependent reaction and ensuring an inert atmosphere discards any effect during SNF dry management. However, at what extent temperature and oxygen concentration would have an impact on the U_3_O_8_ formation is not established, being the available experimental data very scarce. We follow this oxidation in representative ranges of temperature and oxygen concentration of dry storage facilities by using in-situ Raman spectroscopy. The results show that temperature is a more-affecting factor than the oxygen concentration at the studied conditions. Therefore, efforts to limit temperatures would yield more benefits in preserving fuel matrix integrity.

## Introduction

There is an international and increased concern on the Spent Nuclear Fuel (SNF) integrity during SNF management for pre-disposal activities, being the interim dry storage the intermediate step before the final disposal. At present, dry storage is one of the shortest-term solutions for SNF accumulation difficulties in the near future because of the limited space in water-pool, used as a passive heat-removal system for cooling, and the uncertainty of the deep disposal operation schedule, which is the last stage planned. In dry storage conditions, the safety of the fuel must be ensured mainly in front of oxidation, which can occur at high-enough temperature in presence of oxygen, and may possibly lead to a potential mechanism affecting the clad integrity driven by the formation of U_3_O_8_.

Therefore, the formation of U_3_O_8_ and other intermediate oxides from the oxidation of UO_2_, the main constituent of most of the nuclear fuels (thus being often called “Spent Nuclear Fuel matrix”), has received considerable attention through the past decades^1–7^. The study of the different parameters that can affect this reaction, such as temperature and oxygen partial pressure, is of interest for irradiated fuel under operational and accidental scenarios during handling and transport. This has been the origin of regulation limiting the exposure of fuel matrix to temperature and oxidant species^8–12^.

The oxidation of UO_2_ is a well-understood process, which involves a number of intermediate oxides with different characteristics. In the case of unirradiated fuel, these oxides include the generally labelled UO_2+x_, where cubic lattice from the original UO_2_ is still maintained; U_3_O_7_, that implies a change in the structure, obtaining a phase with a tetragonal lattice; and U_3_O_8_, in which lattice changes are again observed, obtaining an orthorhombic oxide. This phase is the final product of the oxidation at the range of temperatures and pressures expected during the fuel storage^4,5^. Compared to UO_2_, U_3_O_8_ entails an increase in volume of around 36%, and comes along with spallation and pulverization of the initial UO_2_ sample^5^. Therefore, the formation of U_3_O_8_ could lead, depending on the condition of a pre-existent failure in the clad, to failure propagation, splitting the clad and even allowing certain release of radioactive material^13–16^.

Due to its practical importance from the nuclear safety point of view, the boundary conditions in current nuclear storage facilities at which U_3_O_8_ does not form are widely studied (i.e. low temperature and lack of oxygen). Other conditions impacting on the formation of U_3_O_8_ have been studied under the circumstances expected during dry storage of the fuel, especially focused on temperature. At the interim storage facilities, the upper limit temperature allowed in inert atmosphere for LWR fuel is 400 °C^9,11,17^, to protect the fuel rod cladding^18^. During normal conditions of storage, there will be a range of temperatures lower than the maximum allowable of 400 °C. That’s why most of the studies regarding the formation of U_3_O_8_ during oxidation of SNF have been conducted at this temperature or below^5,15,19–22^. The huge majority of these experiments have been carried out by using thermogravimetric analysis and flowing air as the oxidant phase.

About the reaction atmosphere, the fuel has been mainly studied after being exposed to air. Investigating the potential oxidation of the fuel matrix at lower oxygen partial pressures, i.e., with specific loading cells being partially inert, might bring to other alternatives to air that might delay or even prevent this oxidation. However, the literature regarding the effect of lower oxygen partial pressures than air on UO_2_ oxidation is scarce. With SNF, Nakamura et al.^23^ oxidized prepared defective fuel rods with burnups ranging from 14 to 30.6 GWd/tU at 200-240ºC in different atmosphere (1%, 5% and 21%O_2_). At 200 °C, they found a decrease of the oxidation rate with the oxygen content and a stabilization of the U_4_O_9_ phase^5,24^. In addition, Kolyadin et al.^25^ studied the oxidation of irradiated samples (burnups between 10.07 and 19.7 GWd/tU) with very low oxygen atmospheres (0.08%, 0.48% and 1.3%O_2_) at temperatures in the range of 100–300 °C. They concluded that the lower the oxygen concentration, the slower the oxidation reaction.

Unirradiated fuel has been studied only by a few authors at different temperatures and atmospheres^2,26–28^. In addition, most of the studies published up to date were conducted by using traditional techniques, mainly thermogravimetry. This technique gives information about the kinetic behavior of UO_2_ oxidation under the specified conditions, as well as the different mechanisms that take place during this reaction by studying the shape of the weight gain curves^5^. This technique measures the “bulk” oxidation of the sample, giving as a result an average O/M value reached by UO_2_ after the experiment. Taking into account the possible security controls at storage facilities, it could be interesting to detect the potential early apparition of U_3_O_8_ and follow the progress of the reaction even if the entire sample is not yet oxidized. Only Olsen et al.^28^ recently studied the oxidation of purified UO_2_ in a wider range of temperatures (100–400 °C) and oxygen partial pressures (0–21%O_2_). They aged the samples in a furnace with controlled atmosphere at different times (2, 25 and 48 h) and characterized ex-situ the final product at each condition by XRD. Nevertheless, more studies covering a complete range of temperatures and oxygen concentrations under the same experimental conditions are needed to support and validate the singular conclusions from each study.

In this work, we study the effect of three temperatures at and below the aforementioned maximum allowable of 400 °C^9,11^ (i.e. 200, 300 and 400 °C) each of one at four different atmospheres (0.1, 1, 10, and 21% O_2_) on the formation of U_3_O_8_, which is the terminal thermodynamic state at these conditions^29^, from a fresh UO_2_ surrogate, whose representativeness is proved in terms of its conservatism in relation to actual spent fuel^30^. In addition, in this study the oxidation at in-situ conditions is measured from really short times (2 min) up to longer times (3 days), depending on the oxidation products obtained at every specific condition.

In this regard, Raman spectroscopy is presented in this work as a non-destructive technique able to meet this purpose. This technique provides a fingerprint to distinguish between chemically similar compounds, as it is the case of uranium oxides. Besides, it gives the possibility to perform in-situ measurements, allowing studying the materials in the real conditions at which they will be in a real storage facility. Raman spectroscopy has been previously used in the characterization of uranium oxides^31–40^. In our laboratory, several identification studies have been carried out^41–44^, and some initial tests have been performed in-situ^41,45^. However, to the best of our knowledge, this is the first time that this technique is employed to study the oxidation of UO_2_ at in-situ conditions, including lower oxygen partial pressures than air. Also, the measurement protocols developed here can be directly applied in the study of irradiated fuel.

Thus, the main goal of this work is to analyze in detail and clarify the impact of changing, in a broad range of conditions, both the temperature and the oxygen concentration in the atmosphere in contact with SNF on the oxidation of the spent fuel matrix.

## Results

The aim of this work is to evaluate the combined effect of the decrease in the O_2_ content and the temperature on formation of the oxidized phases of UO_2_. The method used to study this reaction is based in the analysis of the Raman spectra obtained at in situ conditions (0.1, 1, 10 and 21% O_2_ and temperatures of 200, 300 and 400 °C). These conditions are obtained by using a Linkam stage which has been slightly modified in order to house it to a Raman spectrometer (see “Methods” section).

The Raman spectra of each oxidized UO_2_ phase (i.e. UO_2+x_, U_3_O_7_, U_3_O_8_) is well known^31,32,34,35,38,46–48^ and, as it was confirmed in our previous works^41–45^, this spectroscopy can unequivocally identify each phase at room temperature. At higher temperatures, we already demonstrated that the main features of the spectra corresponding to UO_2_^42^ and UO_2+×_^45^ do not suffer any drastic change, but as expected, all Raman bands downshift in wavenumber and increases its bandwidth with increasing temperature. Raman spectra of U_3_O_7_ and U_3_O_8_ at higher temperatures are evaluated here.

The analysis was carried out as follows: we first establish the criteria to identify the different phases i.e. determine the Raman fingerprint or Raman signature of each phase. After verifying that the characteristics corresponding to the U_3_O_7_ and U_3_O_8_ phases are not modified due to temperature, these Raman signatures are used to analyze the spectra acquired in situ in the different experiments, i.e. the Raman spectra acquired at each time (up to 3000 s, i.e. around 2 days), temperature and O_2_ concentration.

### UO_2_, UO_2+x_ U_3_O_7_ and U_3_O_8_ Raman signatures

**Figure 1:**
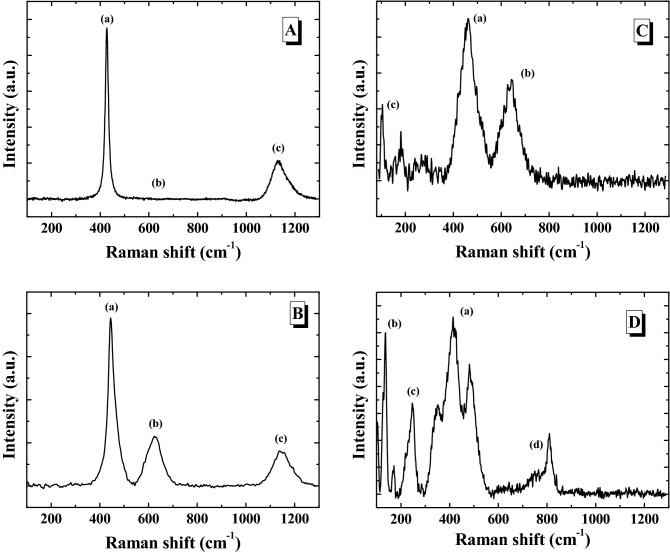
Raman signatures of the different uranium oxides characterized in this work, being (**A**) UO_2_ (**B**) UO_2+x_ (UO_2.10_) (**C**), U_3_O_7_ and (**D**) U_3_O_8_.

The characteristic UO_2_ spectrum shows (see Fig. 1A) the triply degenerate T_2g_ mode at 445 cm^−1^ (a), the LO mode at about 570 cm^−1^ (b), and the first overtone of the L-O mode (2LO) centered about 1140 cm^−1^^31^ (c). Figure 1B shows the spectrum corresponding to UO_2+x_ with x = 0.1. This x value represents the average composition of the sample, probably formed by a mixture of UO_2_ and U_3_O_7_ in the conditions studied here^29^. As is clearly shown in the figure, with the increase in the oxidation the spectrum suffers a broadening and upshifting of the 445 cm^−1^ band (a), an increase in the intensity of the 570 cm^−1^ (b) and a decrease in the intensity of the 2LO band (c) compared with the initial UO_2_ (x = 0). Moreover, the spectrum shows the apparition of a new band near 630 cm^−1^ associated to the oxidation^31,41^. It should be noted that these two bands (570 cm^−1^ and 630 cm^−1^ bands) overlap, giving a broad band centered above 600 cm^−1^, which will be referred in this work as defects band (b)^41^.

The characteristic Raman spectrum of the next oxidized phase U_3_O_7_ is presented in Fig. 1C. It is identified by the two bands corresponding to the T_2g_ mode (a), the band associated to the defects above 600 cm^−1^ (b) and the fading of the 2LO band originally at ~ 1140 cm^−1^^31,41,49^. In this phase, an extra Raman feature located at about 120 cm^−1^ (c) becomes visible, which has been assigned to tetragonal distortions of the cubic lattice of UO_2_, therefore confirming that the spectrum corresponds to U_3_O_7_^31,50^.

Finally, the Raman spectrum of U_3_O_8_ is presented in Fig. 1D. Compared to previous oxidized phases, it is characterized by a completely different pattern. The main Raman feature is the broad triplet in the 300–500 cm^−1^ region (a), associated with the different modes of the U–O stretching in the orthorhombic lattice^46,47^. This mode has been used as a fingerprint for identification of this compound^33^. The Raman spectrum of U_3_O_8_ also presents a quartet of modes between 90 and 150 cm^−1^ (b), an intense peak at 240 cm^−1^ (c), and a broad band around 750 cm^−1^ together with a narrower peak at around 810 cm^−1^ (d). The assignment of these bands is more or less known, although some details are still missing^33,47,51^.

As seen in Fig. 1, the evolution of the Raman spectra with the oxidation can be described as follows: the initial UO_2_ spectrum varies by increasing the intensity of the band located above 600 cm^−1^ and decreasing the intensity of the band at 1140 cm^−1^ (2LO band) when oxidized to UO_2+x_. Thereafter, when U_3_O_7_ is formed, the 2LO band disappears and therefore the spectrum has two intense features at 445 cm^−1^ and above 600 cm^−1^. The change that occurs when the oxidation proceeds up to U_3_O_8_ is evidenced by the triplet of bands in the 300–500 cm^−1^ region.

**Figure 2:**
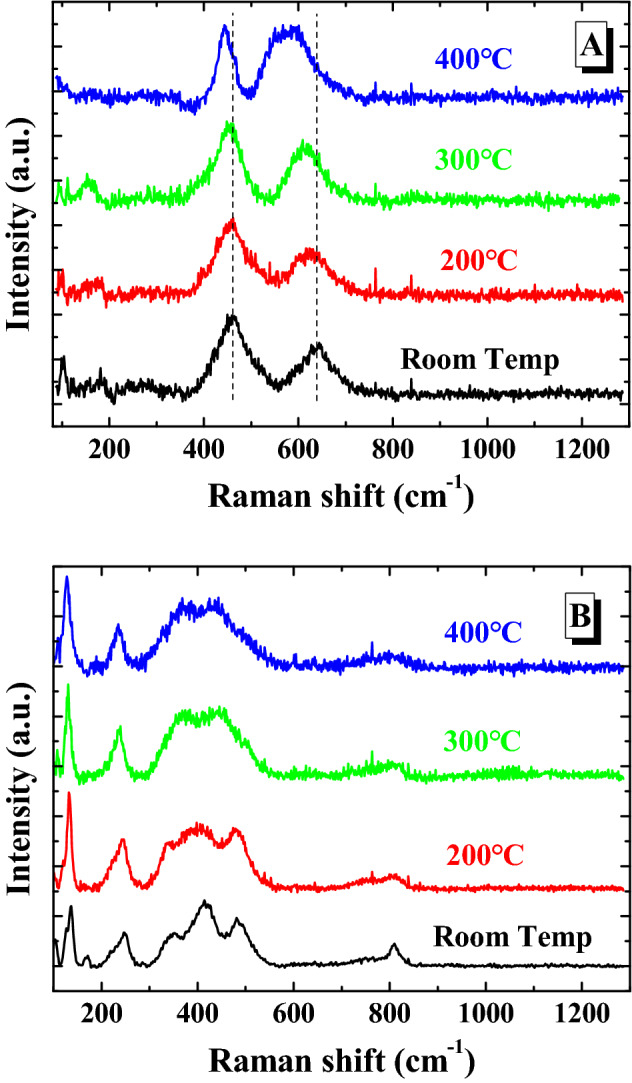
Raman spectra of (**A**) U_3_O_7_ and (**B**) U_3_O_8_ under inert conditions (Ar) as a function of temperature in the region studied in this work.

Once we have stabilized the Raman signature of each phase, let us to check that the effect of the temperature do not modify the Raman fingerprint of the U_3_O_7_ and U_3_O_8_ phases described above. In order to reach this goal, we have measured the U_3_O_7_ as U_3_O_8_ phases as a function of temperature at inert conditions (Ar) (Fig. 2A,B). According to the results, the main Raman modes suffer a slight broadening and shift to lower wavenumbers. Nevertheless, the main spectral features remain constant for the working temperatures, allowing us using them to identify the different oxides not only at room temperature but also at high temperature up to 400 °C. On the one hand, for U_3_O_7_ (Fig. 2A), the disappearance of the 2LO band with respect to the UO_2+x_ spectrum is used. On the other hand, for U_3_O_8_ (Fig. 2B), the presence of the broad signal in the 300–500 cm^−1^ region, although the triplet is not as evident as at room temperature, can still be used for a quick identification of this phase.

### In situ Raman study of the UO_2_ oxidation at different atmospheres and temperatures

By using the Raman signatures described above we have identified the formation of each phase formed at each time (up to 3000 s, i.e. around 2 days) at the testing conditions (0.1, 1, 10 and 21% O_2_ and temperatures of 200, 300 and 400 °C). Next paragraphs show some representative results obtained at different conditions studied in this work. They have been selected in order to present an illustrative picture of the possible scenarios that could be considered in new design options of dry storage facilities. The rest of the results, covering the complete range of experiments, can be found at the supplementary information.

**Figure 3:**
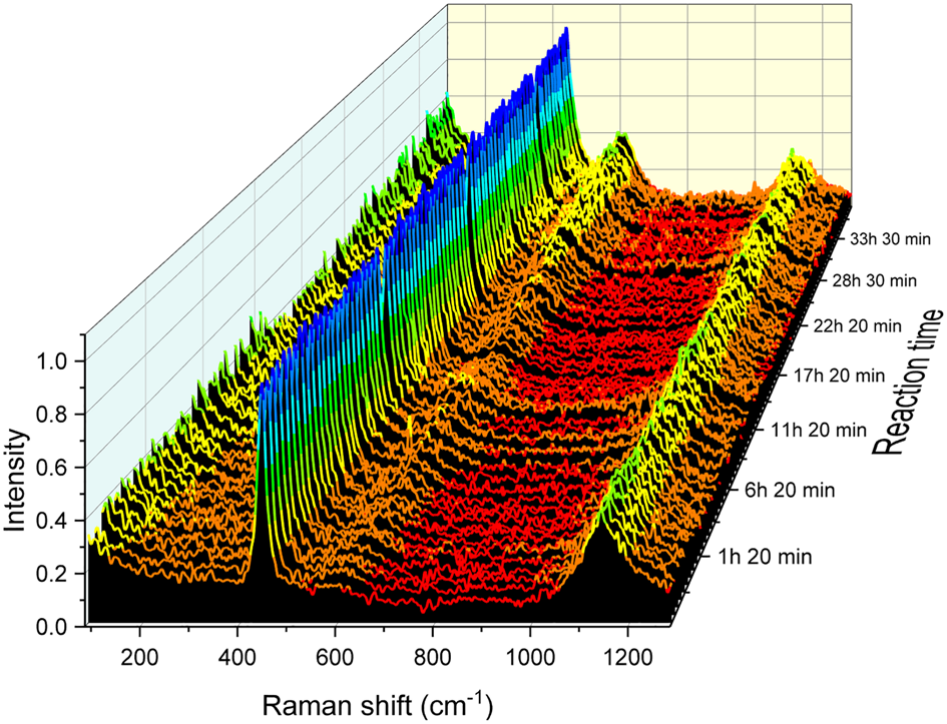
Raman spectra acquired during the experiment at 200 °C and 0.1%O_2_.

Figure 3 shows the spectra as a function of time obtained at the lowest temperature and O_2_ concentration studied (200 °C and 0.1%). In the Figure, the typical features of the hyperstoichiometric UO_2+x_ phase are obtained in the whole time period experimented i.e. three main bands at around 445, above 600 and 1140 cm^−1^ corresponding to the aforementioned T_2g_, “defects band” and 2LO modes. It should be noted that the evolution of the spectra as a function of the oxidation time is demonstrated by the continuous increase in the intensity of the band above 600 cm^−1^ and the decrease in the intensity of the 2LO band.

**Figure 4:**
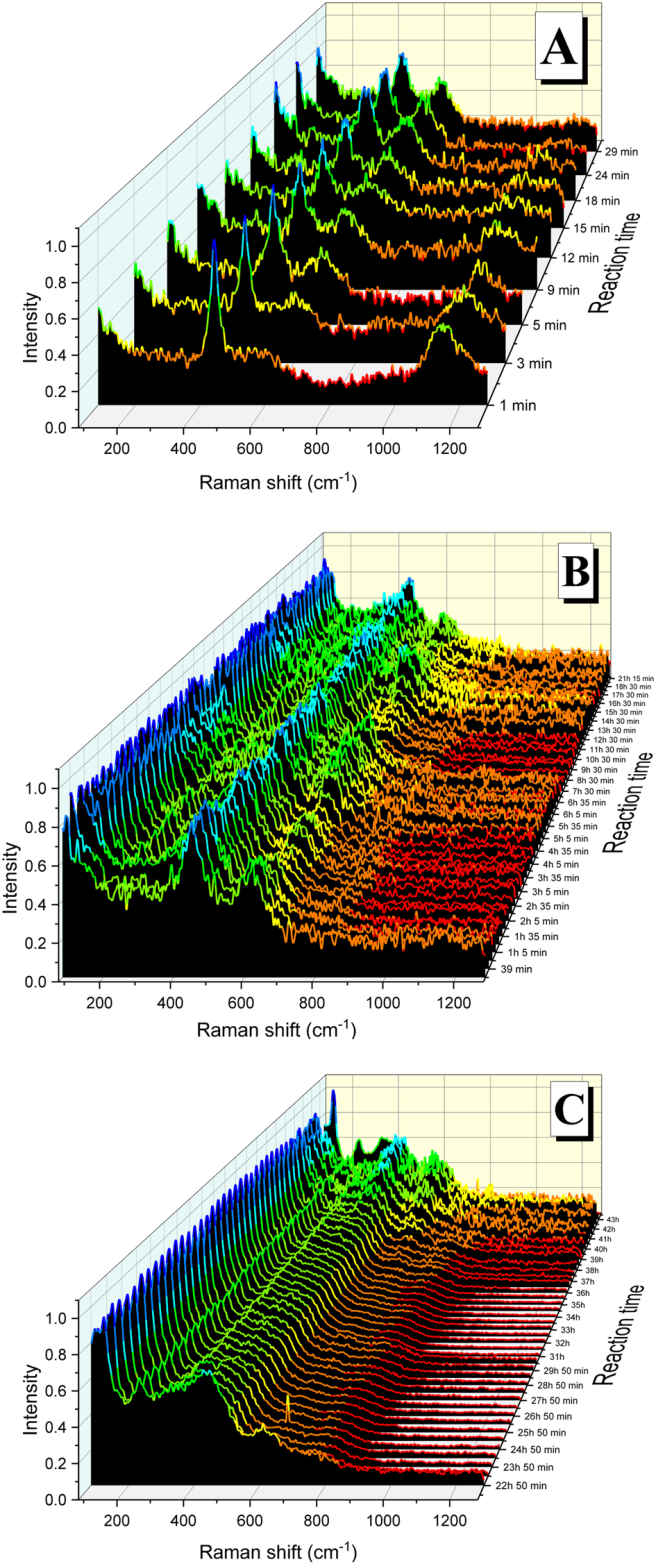
Raman spectra acquired during the experiment at 300 °C and 0.1%O_2_ showing the formation of different oxidized phases from the initial UO_2_: (**A**) UO_2+x_ up to 18 min, (**B**) U_3_O_7_ from 24 min to 21 h and (**C**) U_3_O_8_ from 22 h to the end of the experiment.

At same O_2_ concentration (0.1%) and higher temperature (300 °C) the spectra show the formation of UO_2+x_ up to 18 min (Fig. 4A), note the three bands at around 445, 600 and 1140 cm^−1^. At times close to 24 min the disappearance of the 2LO band (1140 cm^−1^) becomes evident, indicating the formation of U_3_O_7_ (Fig. 4B). This phase prevails up to times of 21 h, after this (at times ranging from 22 to 45 h) the apparition of the trident evidences the formation of U_3_O_8_ (Fig. 4C).

**Figure 5:**
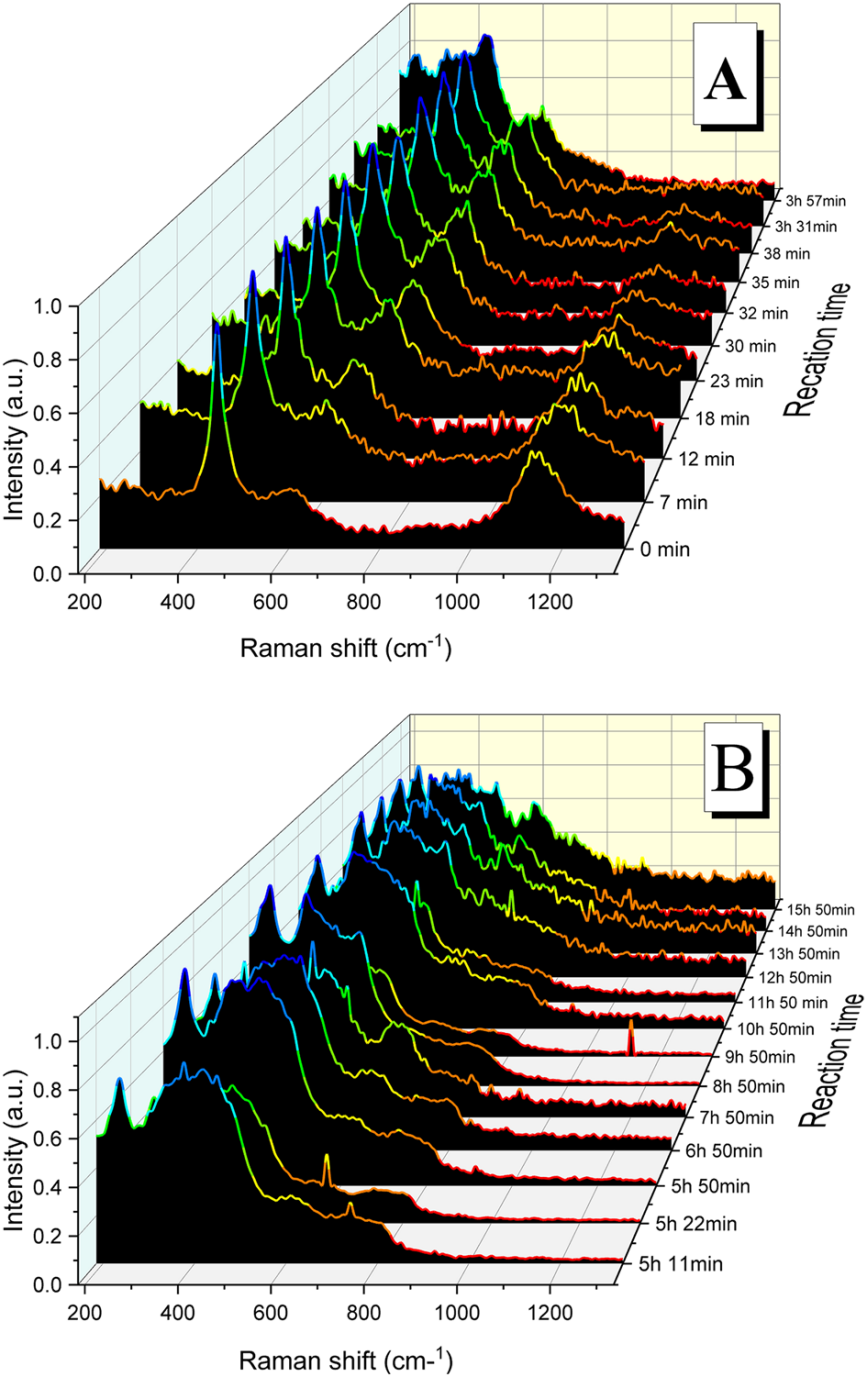
Raman spectra acquired during the experiment at 300 °C and 21%O_2_ showing the formation of different oxidized phases: (**A**) UO_2+x_ and U_3_O_7_ during the first 5 h and (**B**) U_3_O_8_ from 5 h on.

Same three phases (UO_2+x_, U_3_O_7_ and U_3_O_8_) have been detected at 21% O_2_ and 300 °C (Fig. 5). At short times (up to 23 min) the spectra have the three main bands corresponding to the UO_2+x_ phase, then the disappearance of the 2LO band (1140 cm^−1^) evidence the formation of U_3_O_7_ (Fig. 5A), and finally from around 5 h the spectra correspond to the U_3_O_8_ phase (Fig. 5B).

From the spectra acquired at the different experimental conditions, and previously presented, valuable information can be extracted. The spectral differences aforementioned have been associated to the structural changes that occur in UO_2_ when starts to oxidize first to UO_2+x_, maintaining the cubic lattice; then proceeding via U_3_O_7_ with such a distortion that the lattice goes to a tetragonal intermediate; and finally oxidizing up to U_3_O_8_, which is orthorhombic and present completely different Raman bands.

## Discussion

The spectra acquired at the different experimental conditions, and previously presented, have been interpreted in terms of the oxidation degree of UO_2_. The O/U ratio has been estimated according to the evolution of the Raman spectra with time. This assessment has allowed us characterizing the overall reaction and to divide it into three different phases UO_2+x_, U_3_O_7_ and U_3_O_8_. First of all, the initial step of the reaction has been evaluated by determination of “*x*” in the structure UO_2+x_, to confirm the increasing oxygen incorporation to the lattice with time, but not observing a substantial structural change reflected in the spectra. With this purpose, we have used a previous analysis of the Raman spectra as a function of ×^41^, taking also into account the temperature correction factor^45^, as shown in Eqs. (1) and (2).

1 $${\Delta \mathrm{v}}_{630}\left({\mathrm{cm}}^{-1}\right)=-\left(610\pm 60\right)x-\left(0.049\pm 0.003\right)T(\mathrm{K}), ({\mathrm{I}}_{630}/{\mathrm{I}}_{445}<0.24),$$

2 $${\Delta \mathrm{v}}_{630}\left({\mathrm{cm}}^{-1}\right)=-\left(90\pm 20\right)x-\left(0.049\pm 0.003\right)T(\mathrm{K}), (0.27<{\mathrm{I}}_{630}/{\mathrm{I}}_{445}<0.24).$$

In Eqs. (1) and (2), ${\Delta \mathrm{v}}_{630}$ is defined as ${\Delta \mathrm{v}}_{630}={\mathrm{v}}_{630}-{\mathrm{v}}_{\mathrm{int}}$, being ${\mathrm{v}}_{630}$ the Raman shift of the band associated to the increase in the oxidation degree^31,35,41,51–53^, and ${\mathrm{v}}_{\mathrm{int}}$ the Raman shift of the linear fit intercept. To obtain both Raman shifts, a detailed profile analysis has been carried out in every single spectrum. Central position of the spectral bands was obtained by the second derivative method^54^. The use of two equations to calculate *x* responds to the distinct regions observed in this transition, as reported by Elorrieta et al.^41^.

Secondly, a second step in the oxidation reaction is defined, where the intermediate U_3_O_7_ phase is mainly obtained and interpreted as a stabilization phase of the reaction before reaching the final oxidation product. This phase has been associated with an O/U between 2.25 and 2.40, according to different values reported in literature^7,31,55,56^. The Raman spectrum of this phase shows two strong bands (the T_2g_ mode at ~ 445 cm^−1^ and the oxygen-clusters-related ~ above 600 cm^−1^ band), and the lack of the 2LO mode, which makes it distinct from the UO_2_ and the UO_2+x_ spectrum^31,38,41,57^. Finally, a third step in the reaction is defined by the presence of acquired spectra corresponding to the U_3_O_8_ phase, which is associated with the final of the oxidation process (O/U = 2.67).

**Figure 6:**
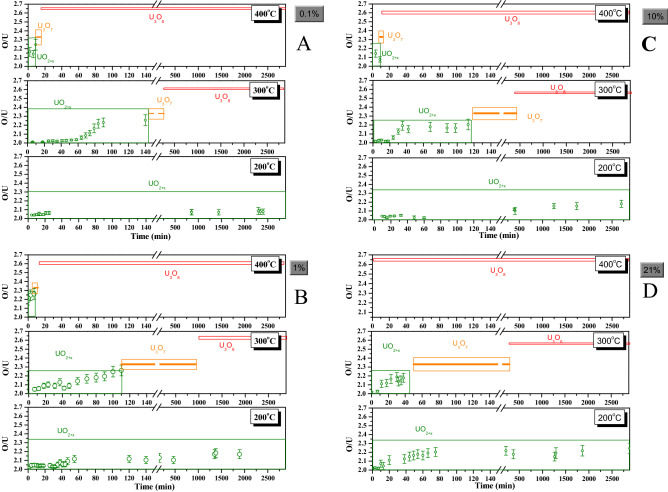
O/U ratio vs reaction time in the different temperatures and oxygen partial pressures studied.

By following these guidelines, the overall progress of the oxidation in the different conditions studied is presented in Fig. 6, in terms of the O/U ratio vs reaction time. The results have been plotted distinguishing temperatures (200, 300 and 400 °C) and oxygen partial pressures at which the oxidation has been carried out (0.1%, 1%, 10% and 21%O_2_).

At 200 °C, only the UO_2+x_ phase is observed, regardless of the oxygen concentration, in the whole duration of the experiments (approximately 2 days per temperature and oxygen concentration). The effect of the limited availability of oxygen in this low temperature is the extent of the UO_2+x_ phase, i.e. the O/U value. From Eqs. (1) and (2), we have calculated the O/U, obtaining that with 0.1%O_2_ is O/U = 2.07 ± 0.03. This value grows with oxygen concentration, resulting in O/U = 2.17 ± 0.05 with 1%O_2_; O/U = 2.18 ± 0.05 with 10%O_2_; and O/U = 2.24 ± 0.06 with air (21%O_2_) (Fig. 6A–D, respectively). Thus, no formation of higher oxides has been detected in any case. In addition, it should be highlighted that the effect of a higher presence of the oxidant is to accelerate the first step of the reaction with time. This effect has been previously reported as a consequence of the diffusion mechanism in the first part of the oxidation of UO_2_^1,4,7,58^. In fact, Blackburn et al.^2^ measured the oxidation of unirradiated UO_2_ at 200 °C covering a range of oxygen concentrations from 0.07 to 100%O_2_. They reported a lack of effect on the oxidation degree between 20 and 100%O_2_, but stated that a decrease of the oxidation occurred in the 0.07–20%O_2_ range when the oxygen concentration was low, being consistent with the results of this work.

On the other hand, at 400 °C the U_3_O_8_ phase is prevalent, independently of the oxygen concentration. In the most extreme case, i.e. 21%O_2_, only U_3_O_8_ is observed even at the very beginning of the experiment, giving an idea of the quickness of the oxidation at these conditions. These data could be taken into account in new designs for nuclear fuel storage facilities given the risk of fuel exposure to air at this temperature. With lower oxygen partial pressures, it is possible to observe the lesser oxidized phases UO_2+x_ and U_3_O_7_. However, the temporal frame of the reaction is very short, being U_3_O_8_ the only oxide observed from 20 min even with the lowest oxygen concentration, 0.1%. Earlier in this case, UO_2+x_ spectra, with the *x* value continuously increasing, are acquired during approximately 10 min. At this point, the transformation to U_3_O_7_ seems to happen, obtaining a couple of spectra corresponding to this phase during the period 10–20 min (Fig. 6A). The increase of the oxygen presence results in a shortening of the time where the lower oxides are observed, obtaining U_3_O_8_ spectra from around 15 min with 1%O_2_ and from around 10 min with 10%O_2_ (Fig. 6B,C, respectively).

This tendency, maintained through all the oxygen concentrations studied, emphasizes the importance of the fuel temperature, and points out to put efforts on limit the fuel temperature rather than partially inertize (above 0.1%O_2_), which would yield more benefits in preserving fuel matrix integrity. These results, agreeing with published studies using other techniques, such as those reported by Olsen et al.^28^, who by using XRD observed the formation of a 100% of U_3_O_8_ at 400 °C within 2 h when any oxygen is present (the lack of a measurement at shorter times in the referenced study makes a deep comparison of the early apparition of U_3_O_8_ impossible). This behavior is comparable to the oxidation of irradiated fuel, as demonstrated in the study by Hastings et al.^22^. They studied the oxidation of CANDU fragments irradiated at low burnup levels (8 GWd/tU) in air, in the same temperature range than this work (200–400 °C). At 200 °C, they did not observe any weight change during 24 h of experiments. However, at 400 °C, they reported a severe sheath splitting due to the formation of U_3_O_8_ in less than 24 h.

At intermediate temperatures, i.e. 300 °C, the formation of U_3_O_8_ is not as fast as in the previous case, but this phase is still clearly observed from a time around 24 h in all the cases. At 300 °C, the effect of decreasing the oxygen concentration is to delay the formation of U_3_O_8_, extending the temporal frame when UO_2+x_ and U_3_O_7_ are present. Thus, at the lowest oxygen concentration, 0.1%O_2_, UO_2+x_ spectra are acquired during the first 140 min approximately, with increasing *x*. From that moment, U_3_O_7_ becomes visible and appears until around 10 h of experiment, when U_3_O_8_ becomes principal. When increasing the oxygen partial pressure, the observed results indicate that the time range where UO_2+x_ is observed is increasingly shorter, and U_3_O_7_ phase appears earlier. However, the final result of every experiment at this temperature is the formation of U_3_O_8_ at a time around 16 h for the lowest oxygen concentrations, and even earlier when the amount of oxygen present is higher. This would suggest that the temperature dependence over oxygen content on the oxidation of UO_2_ is therefore more evident at relatively high temperature, especially from 300 °C. In fact, it seems that once that a certain threshold temperature for the formation of U_3_O_8_ is exceeded, this phase will be formed even with low oxygen availability (as low as 0.1%). Therefore, the effect of this oxygen decrease will be to delay the presence of U_3_O_8_.

From the analysis of Fig. 6D, one may note that the observed change in the O/U ratio resembles the typical sigmoidal behavior characteristic of the oxidation. In a similar time scale, this pattern has been found in thermogravimetric analysis of UO_2_ oxidation^7^. This fact reinforces the use of Raman as a suitable technique for studying this reaction, and validates the conclusions obtained here.

## Conclusions

In this work, representative oxidation experiments with the aim to determine the combined effect of partial inertization and temperature on the formation of U_3_O_8_ are performed in order to be considered in new design options of fuel storage facilities. Specifically, we study the impact of the oxygen partial pressure (0.1 to 21%O_2_) and temperature (200–400 °C) in the oxidation of fresh UO_2_ (labelled as P3 in the reference^30^). With this purpose, Raman spectroscopy has been selected as a suitable technique, given first its capacity to analyze the oxidized compounds of UO_2_, second its application at in-situ conditions to follow the formation of the oxidation products in a very long time frame (from min up to days), and third this protocol could be implemented in the analysis of irradiated nuclear fuel.

At 200 °C, no quantitative oxidation of UO_2_ to higher oxides has been detected, neither U_3_O_7_ nor U_3_O_8_, at any of the different atmospheres studied. Given the conservativeness of fresh fuel compared to irradiated fuel, this temperature ensures a low oxidation of SNF matrix that can be considered as a “first barrier” for the spent fuel dry management activities, regardless of the content of oxygen at which the fuel may be exposed.

On the other hand, at 400 °C the oxidation of UO_2_ is extremely fast whatever the oxygen concentration may be. In fact, although the lower oxygen slows down the reaction, even at oxygen concentrations as low as 0.1%, the formation of U_3_O_8_ occurs within 2 h. The larger volume of this phase could entail stresses on the SNF clad, which is the second barrier to prevent radioactive material release.

At intermediate temperatures, i.e. 300 °C, the decrease of the formation of U_3_O_8_ is clearly observed, confirming the predominant effect of the temperature over the oxygen concentration at which UO_2_ is exposed. However, U_3_O_8_ is detected within 24 h for all oxygen concentrations studied.

All in all, the results of this work indicate that temperature is the main factor of the oxidation of fuel while the oxygen concentration of the atmosphere, up to 0.1%O_2_, only affects the reaction rate without preventing it to occur. As a consequence of the present work it could be noticed that, in order to avoid the oxidation of the fuel matrix to U_3_O_8,_ keeping the fuel at low temperatures could be very effective. On the other hand, the control of the oxygen-content (above 0.1%O_2_) in the atmosphere where the UO_2_ is exposed is less effective.

Finally, in order to extrapolate these conclusions to other conditions, further research not only studying other intermediate temperatures, but also other characteristics of the starting materials and involving other characterization techniques will be carried out.

## Methods

The fuel analogues studied consisted of powdered UO_2_ samples coming from monoliths of unirradiated sintered UO_2_ pellets that were crushed into powder in a Mixer Mill MM 400 (Retsch). The resulting powder was used with no further sieving treatment, giving place to a material with a wide particle size distribution (average particle size 5.64 ± 0.07 µm, but with particle’s sizes ranging from ~ 1 µm up to ~ 10 µm). Specific surface area of the material was measured by applying the BET method (with N_2_) and was found to be 0.18 ± 0.01 m^2^ g^−1^. Initial O/U of the substrate is 2.002 ± 0.001. This material has been characterized in detail in our laboratory (labelled as P3 in Ref.^30^).

The gases used in this study with different oxygen concentration were supplied by Air Liquide (France). These gases comprised a mixture of oxygen and nitrogen in different proportions, giving place to three special mixtures as follows: 0.1%O_2_–99.9%N_2_; 1%O_2_–99%N_2_; 10%O_2_–90%N_2_. Finally, synthetic air was also provided by Air Liquide, with a licensed composition of 20 ± 1%O_2_, being the rest N_2_. An extra gas line of Ar is used when inert conditions are required. A certified purity higher than 99.999% is applied for all the gases.

Raman spectra were acquired by means of a Horiba LabRam HR Evolution spectrometer (Jobin Yvon Technology). A red He–Ne laser beam (λ = 632.8 nm) was focused onto the sample through the 50 × objective of an Olympus BX41 microscope. The scattered radiation was then collected with the same objective, dispersed with a 600 grooves/mm grating, and finally detected with a Peltier-cooled CCD detector (256 × 1024 pixels). For the in-situ measurements, the sample was placed inside a Linkam THMS600 temperature-controlled pressure stage. This stage allows maintaining the sample in a closed environment and focusing the incident radiation through a central overture in order to acquire the spectra. The sample temperature is controlled by using a T95-Linkpad temperature controller, which provides temperature stability better than 0.2 °C. Atmospheric environment can be selected changing the gas tubes in a quick-fit.

The experimental work has consisted, first, of placing the sample inside the Linkam stage and closing the lid assembly. After overnight inertization of the chamber with a low flow of Ar gas in order to remove the oxygen, the sample is heated at 10 °C min^−1^ up to the experimental temperature (i.e. 200, 300 or 400 °C), still with the Ar atmosphere to prevent undesirable oxidation. Once the chosen temperature is reached, it is maintained during 30 min to allow thermal stabilization of the sample. After that, the gas is switched to the specific oxidant gas, depending on the experiment (i.e. 0.1, 1, 10 or 21%O_2_). Raman spectra are acquired continuously at random locations of the sample surface within typically 50 s of acquisition and 2 accumulations in the 100–1300 cm^−1^ range. These acquisition conditions have been confirmed as low enough to not oxidize the samples by local heating^41^.

## Supplementary Information

Supplementary Information.

## Data Availability

The datasets generated during and/or analyzed during the current study are available from the corresponding author on reasonable request.
