# Supplementary material for: Exploring the impact of temperature and oxygen partial pressure on the spent nuclear fuel oxidation during its dry management
Source: Sci Rep. 2023 Feb 3;13:1966. doi: 10.1038/s41598-023-29265-w (PMC9898559; doi:10.1038/s41598-023-29265-w)
Supplement: Supplementary file 1 — Supplementary Information. [file 41598_2023_29265_MOESM1_ESM.pdf]

SUPPLEMENTARY INFORMATION

Exploring the impact of temperature and oxygen partial pressure on the spent nuclear fuel oxidation during its dry management

AUTHORS NAMES AND AFFILIATIONS

A. Milena-Pérez[1], L.J. Bonales[1], N. Rodríguez-Villagra[1], H. Galán[1]*

Centro de Investigaciones Energéticas, Medioambientales y Tecnológicas (CIEMAT). Avda. Complutense 40, 28040, Madrid (Spain).

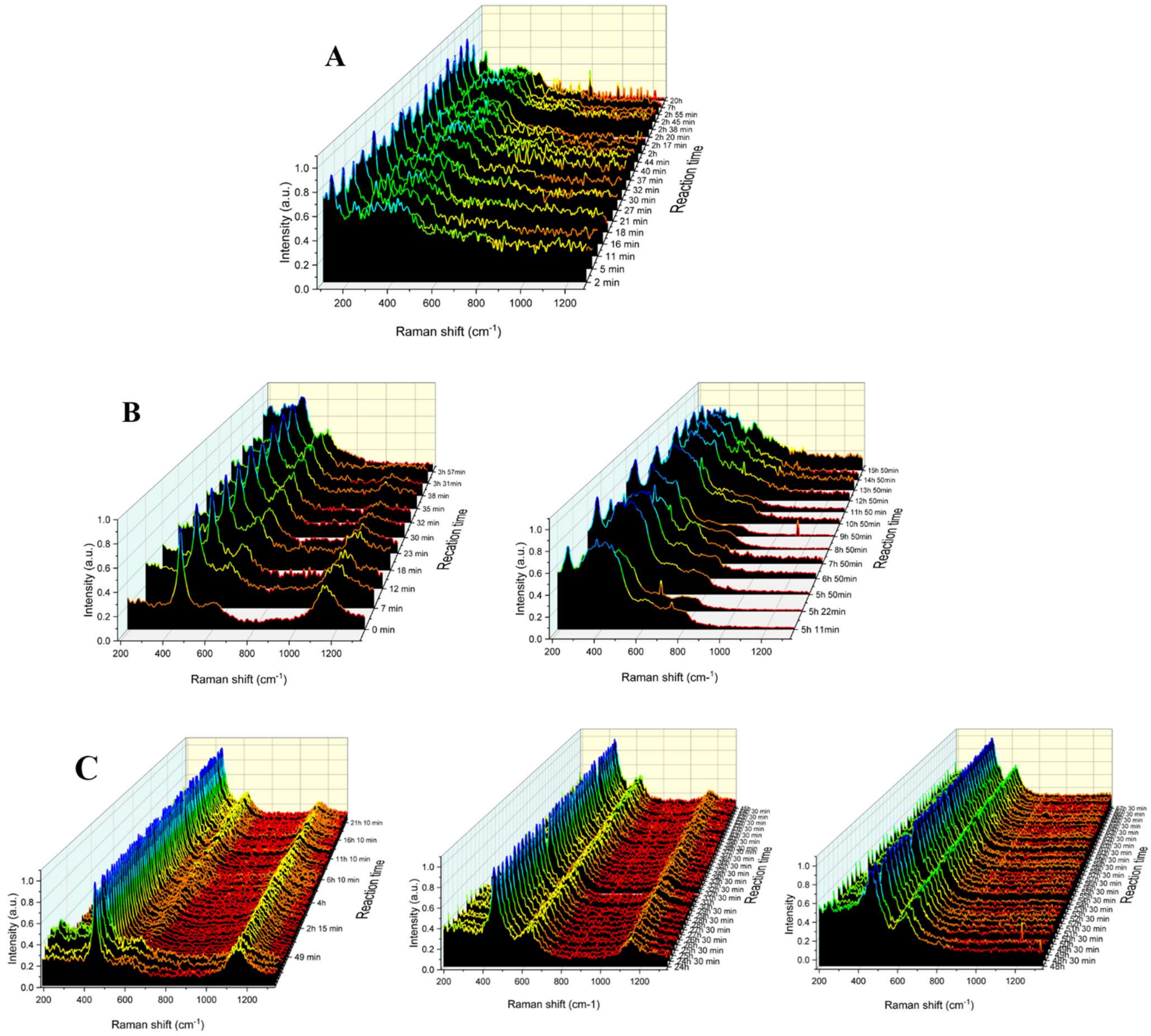

Figure S1. Raman spectra obtained at 21% of O_2 at 400ºC (A), 300 ºC (B) and 200 ºC (C).

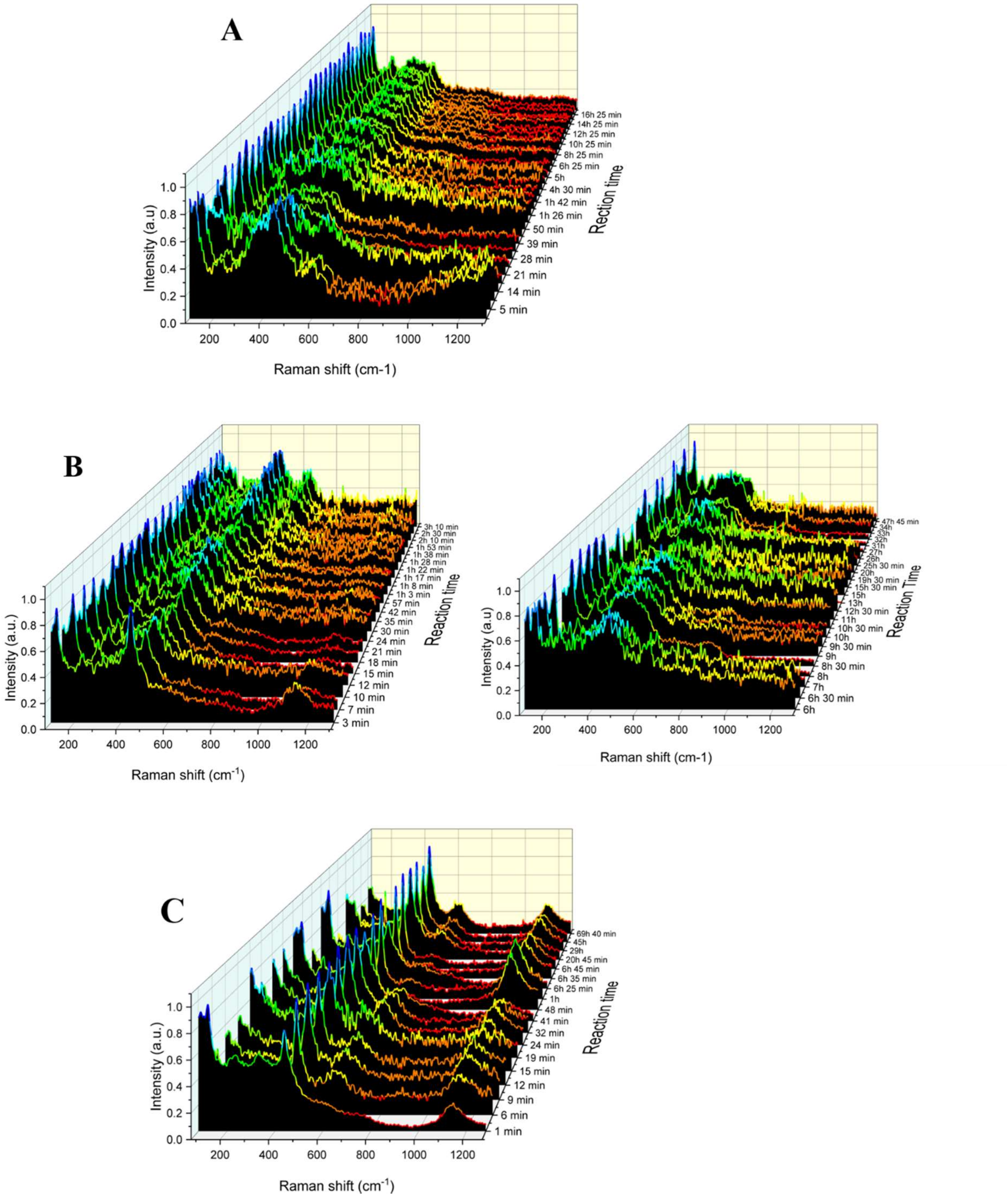

Figure S2. Raman spectra obtained at 10% of O_2 at 400ºC (A), 300 ºC (B) and 200 ºC (C).

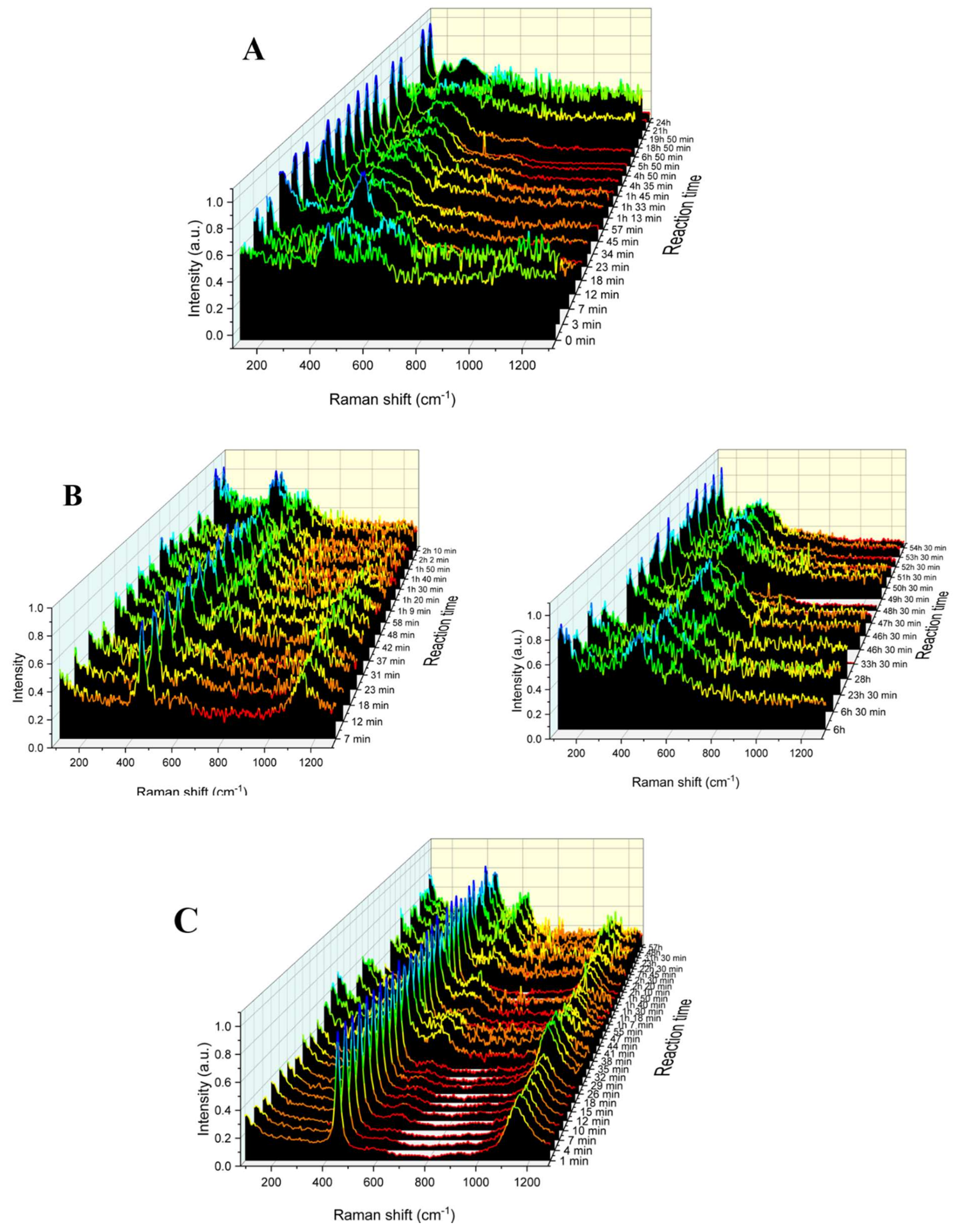

Figure S3. Raman spectra obtained at 1% of O_2 at 400ºC (A), 300 ºC (B) and 200 ºC (C).

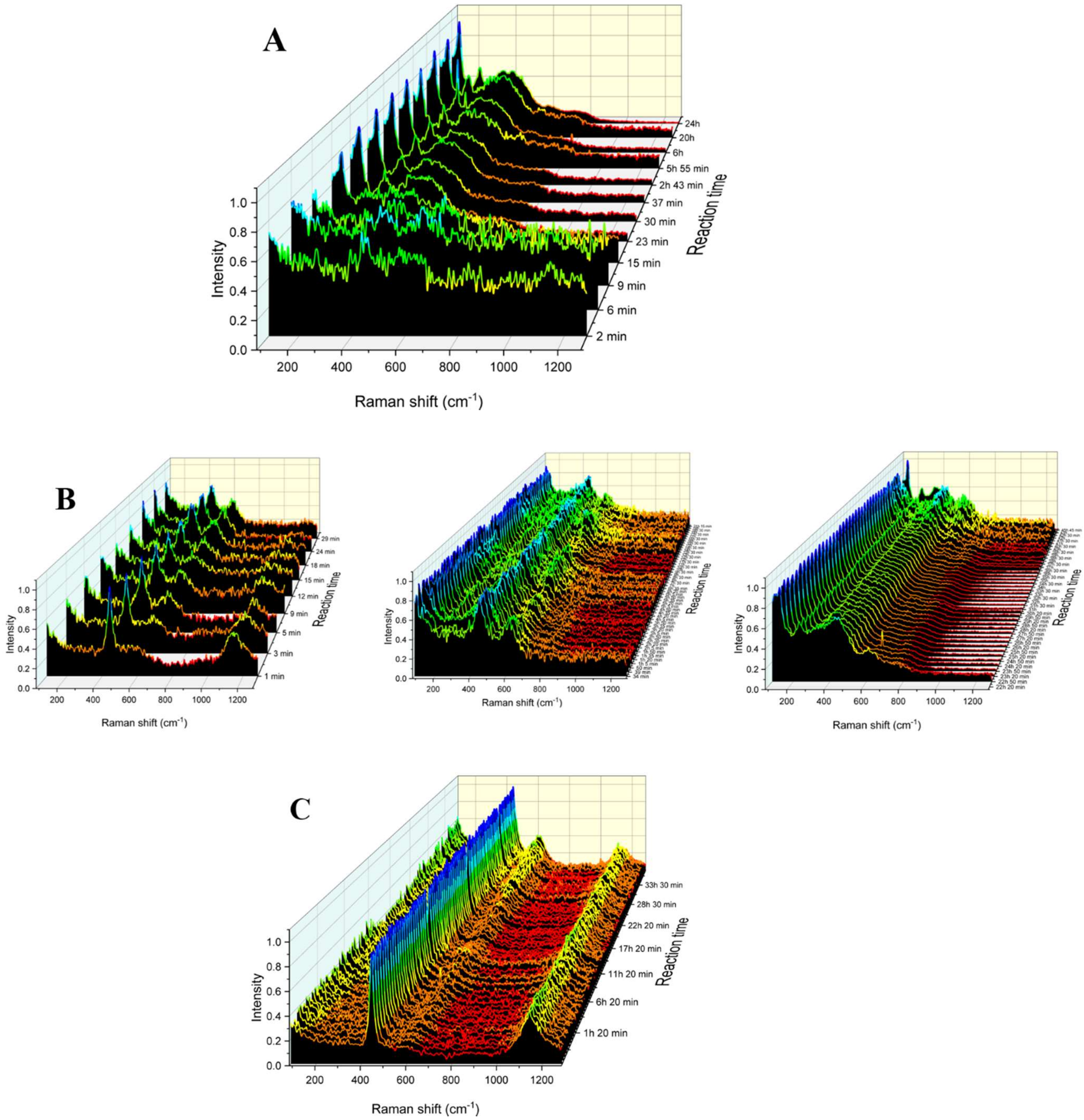

Figure S4. Raman spectra obtained at 0.1% of O_2 at 400ºC (A), 300 ºC (B) and 200 ºC (C).
